# Benefits of Mobile Apps for Cancer Pain Management: Systematic Review

**DOI:** 10.2196/17055

**Published:** 2020-01-23

**Authors:** Caiyun Zheng, Xu Chen, Lizhu Weng, Ling Guo, Haiting Xu, Meimei Lin, Yan Xue, Xiuqin Lin, Aiqin Yang, Lili Yu, Zenggui Xue, Jing Yang

**Affiliations:** 1 Department of Pharmacy Fuqing City Hospital Fuzhou China; 2 School of Pharmacy Fujian Medical University Fuzhou China; 3 Department of Pharmacy Xiamen Maternity and Child Care Hospital Xiamen China; 4 Department of Pharmacy Fujian Medical University Union Hospital Fuzhou China

**Keywords:** mobile apps, cancer pain, meta-analysis, instant messaging

## Abstract

**Background:**

Pain ratings reported by patients with cancer continue to increase, and numerous computer and phone apps for managing cancer-related pain have been developed recently; however, whether these apps effectively alleviate patients’ pain remains unknown.

**Objective:**

This study aimed to comprehensively evaluate the role of mobile apps in the management of cancer pain.

**Methods:**

Literature on the use of apps for cancer pain management and interventions, published before August 2019, was retrieved from the following databases: MEDLINE, Embase, Cochrane, CINAHL, Scopus, and PsycINFO. The effects of apps on cancer pain were evaluated using RevMan5.3 software, and the rates of adverse drug reactions were analyzed using the R Statistical Software Package 3.5.3.

**Results:**

A total of 13 studies were selected for the analysis: 5 randomized controlled trials (RCTs), 4 before-after studies, 2 single-arm trials, 1 prospective cohort study, and 1 prospective descriptive study. The 5 RCTs reported data for 487 patients (240 patients in the intervention group and 247 patients in the control group), and the remaining studies reported data for 428 patients. We conducted a meta-analysis of the RCTs. According to the meta-analysis, apps can significantly reduce pain scores (mean difference [MD]=–0.50, 95% CI –0.94 to –0.07, I^2^=62%, *P*=.02). We then used apps that have an instant messaging module for subgroup analysis; these apps significantly reduced patients’ pain scores (MD=–0.67, 95% CI –1.06 to –0.28, I^2^=57%, *P*<.01). Patients using apps without an instant messaging module did not see a reduction in the pain score (MD=0.30, 95% CI –1.31 to 1.92, I^2^=70%, *P*=.71). Overall, patients were highly satisfied with using apps. Other outcomes, such as pain catastrophizing or quality of life, demonstrated greater improvement in patients using apps with instant messaging modules compared with patients not using an app.

**Conclusions:**

The use of apps with instant messaging modules is associated with reduced pain scores in patients with cancer-related pain, and patient acceptance of these apps is high. Apps without instant messaging modules are associated with relatively higher pain scores. The presence of an instant messaging module may be a key factor affecting the effect of an app on cancer pain.

## Introduction

### Cancer Pain Management

According to the 2018 global cancer statistics, there were approximately 18.19 million new cases of cancer [1]. The number of new cancer cases is increasing rapidly every year and is expected to exceed 20 million by 2030 [2]. Further, the incidence of persistent cancer pain during treatment has also increased [3]. According to reports, approximately 69% of patients with cancer worldwide experience pain during their daily activities, which may have serious psychosocial consequences, including anxiety and depression [4].

Due to differences in treatment levels, cancer pain that is not adequately controlled is still widespread in developing countries [5]. According to reports, only 25% of patients with advanced cancer have pain that can be relieved, especially out-of-hospital patients, who have a lower pain relief rating than patients in the hospital. This is primarily caused by disjointed management of patients after discharge, resulting in reduced patient compliance, poor control of side effects, and outbreaks of pain [6].

### Apps for Cancer Pain Management

With the development of the Internet, the number of health-related apps is growing rapidly, including apps for monitoring and managing diseases [5,7-9]. Apps for pain management are also gradually entering the market [10-12]. There are currently 283 pain-related apps in China and abroad, but only 8.2% of these apps include medical professionals in the development process, and none are scientifically proven to be effective. Therefore, whether apps can improve pain relief for patients remains unknown. Among the available pain apps, none are comprehensive for pain management, and most only contain a pain diary module [13,14].

### Aim of the Study

It is not clear whether using an app to manage cancer pain can improve pain relief rating or which module type is the most effective in terms of app usability and pain management. Therefore, we conducted a systematic review and meta-analysis of studies of apps intended to manage cancer-related pain to explore whether these apps can improve pain relief ratings for patients with cancer and identify which modules increase the app’s effectiveness for managing pain.

## Methods

### Literature Search

Eligible studies were identified by searching Medline, Embase, Cochrane, CINAHL, psycINFO, and other relevant databases, and the results were combined using the literature traceability method. Searches were conducted on August 1, 2019. Search keywords included “cancer pain+,” “cancer pain management+,” “mobile phone,” “+,“ ”Mobile Devices,“ ”Mobile Apps,“ and ”mHealth.“ Search strategies are detailed in Multimedia Appendix 1. Only articles written in English were considered. There were no restrictions regarding publication date.

### Inclusion and Exclusion Criteria

#### Inclusion Criteria

Studies were included when they (1) focused on patients with cancer pain, (2) involved an intervention using apps downloaded and registered on either a mobile phone or computer for the management of cancer pain, (3) used the numeric rating scale to assess pain, (4) included patients that were followed up for more than a week, and (5) reported the results in English.

#### Exclusion Criteria

Studies were excluded when (1) the study type was a review, model study, literature review, or conference summary; (2) the results of the study used scales other than the numeric rating scale or did not report the pain score; (3) the study intervention was a telephone conversation; or (4) the study was a duplicate report.

### Document Screening and Data Extraction

All reference titles and abstracts were initially screened for relevance by two reviewers. Afterward, full-text analysis for eligibility was independently performed by Caiyun Zheng and Lizhu Weng. Disagreements were resolved by discussion and consensus or third-party arbitration.

The required data were extracted by a researcher using a literature data extraction table, and another researcher confirmed the accuracy and authenticity of the data. The extracted content included study information (research topic, author, date), baseline characteristics of the study subjects (sample size, median age), specific details of the app intervention, pain scores for up to 12 months of intervention, and other outcome indicators (quality of life, side effects).

### Literature Quality Evaluation

Risk assessment of the included RCTs was performed using the Cochrane risk of bias tool based on the Cochrane Systematic Review Manual's literature evaluation criteria.

### Statistical Analysis

Meta-analysis of RCTs was performed using RevMan5.3 software. Heterogeneity was assessed using the chi-squared test, and quantitative analysis was performed using I^2^. Values of *P*≥.05 and I^2^ ≤50% were considered to represent no heterogeneity, and a fixed-effect model was used. If *P*<.05 and I^2^>50%, a random-effects model was used, followed by subgroup analysis to identify the cause of heterogeneity [15].

## Results

### Search Results

**Figure 1:**
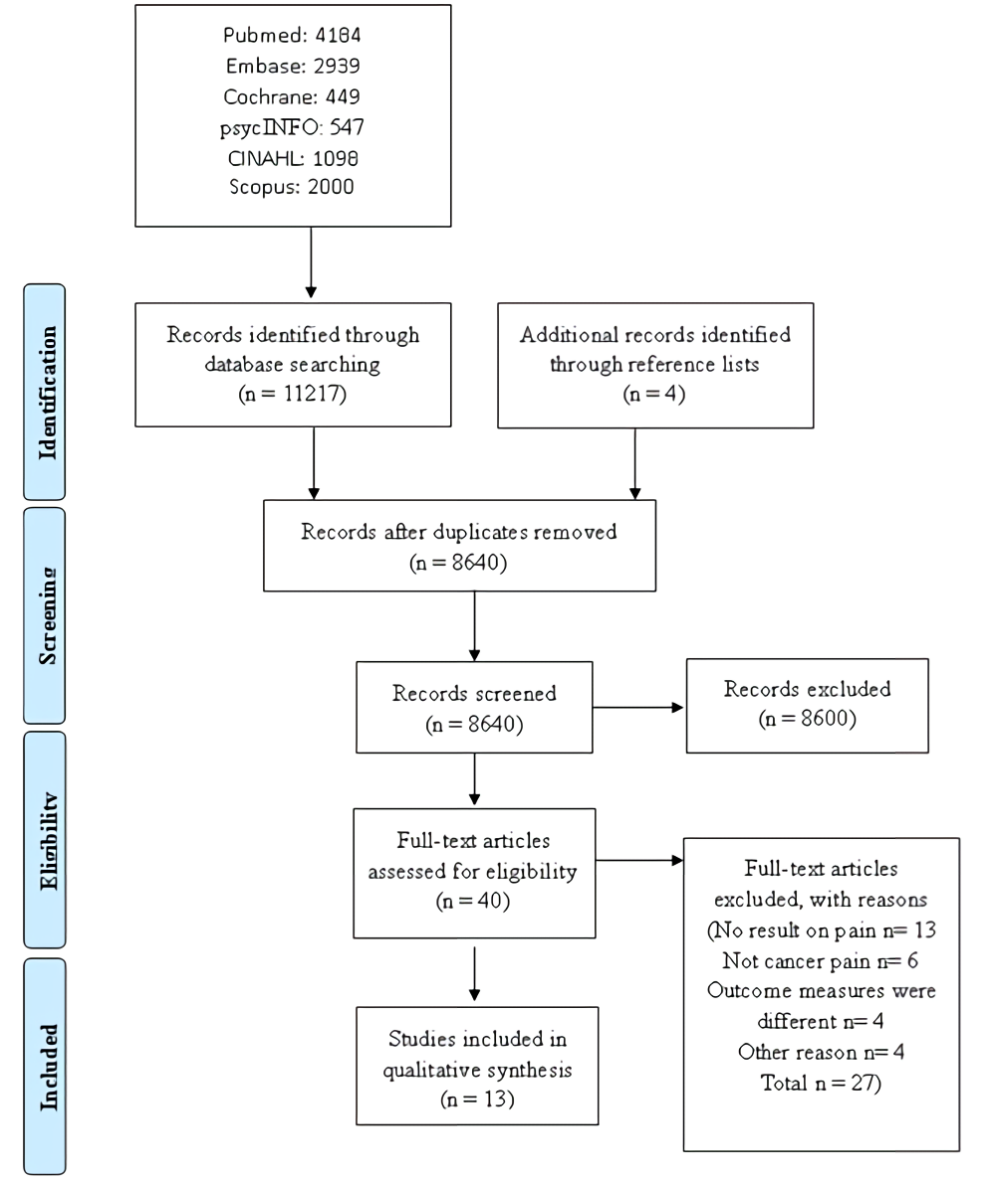
Literature screening and selection flow chart.

A total of 11,217 articles were retrieved from the systematic literature search, and 4 articles were retrieved by other means, totaling 11,221 articles. After removing duplicate studies, the remaining 8640 were screened. After reading 40 eligible full-text articles, 27 were excluded, and 13 were selected [16-28]. The systematic search results are shown in Figure 1.

### Basic Characteristics and Quality Evaluation of the Literature

**Figure 2:**
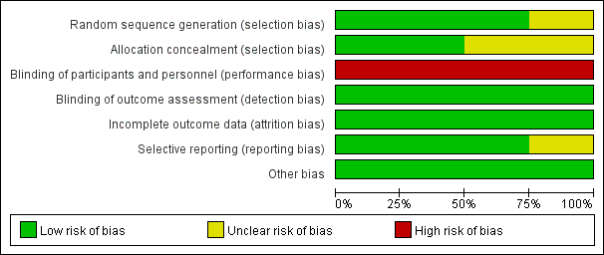
Bias risk map from the Cochrane systematic evaluation method to evaluate the quality of the included randomized control trials.

The 5 RCTs included a total of 487 patients: 240 patients in the intervention group and 247 patients in the control group. The remaining studies reported data for 428 patients. General information from the studies is shown in Table 1. The Cochrane systematic evaluation method was used for quality evaluation, and overall, the included literature had a low risk of bias, as shown in Figure 2.

**Table 1:** Summary of the characteristics of the included studies.

Study	Study design	Number of participants	Follow-up (weeks)	Age (years), mean (SD)	Description of modules within the app	Instant messaging module
Yun, 2012 [17]	RCT^a^	273	12	Unknown	Five modules: self-assessment and graphic reports, health advice and online education, enhanced and short message services, caregiver monitoring and support, monitoring by a health professional	Yes
Somers, 2016 [23]	RCT	23	1	60.00 (11)	Skype	No
Sun, 2017 [21]	RCT	46	2	67.50 (Unknown)	Four modules: life quality self-evaluation, cancer pain self-evaluation, real-time messaging, standard medication	Yes
Smith, 2018 [28]	RCT	87	18	56.70 (8.7)	Four modules: Web-based content, required activities including attending one online introductory group meeting, viewing videos to complete cognitive reframing, mind-body exercises	No
Yang, 2019 [16]	RCT	58	4	52.53 (8.78)	Four modules: pain education, consultation, cancer pain self-evaluation, soothing music	Yes
Somers, 2015 [18]	BAS^b^	25	1	53.88 (12.59)	Videoconferencing on a tablet computer, eg, Skype	No
Jibb, 2017 [24]	BAS	40	4	14.20 (1.7)	Questionnaires, real-time self-management recommendations, email alerts	Yes
Bae, 2017 [26]	BAS	100	4	57.00 (Unknown)	PHR^c^ data gathering, PHR gateway, Web service	No
Lengacher, 2017 [25]	BAS	13	6	57.00 (9)	iBooks on an iPad	No
Stinson, 2015 [22]	Prospective, descriptive study	92	2	13.10 (2.9)	Assessment items	No
Oldenmenger, 2017 [27]	Prospective, cohort study	48	6	59.00 (11)	Three modules: a pain diary, eConsult, patient education	Yes
Dorfman, 2018 [19]	Single-arm pilot study	20	Unknown	57.85 (11.72)	Daily access to session content, video clips modeling coping skills, stories about pain experiences from example participants, other materials (eg, relaxation audio)	Yes
Parks, 2019 [20]	Prospective, single-arm study	90	12	55.10 (8.7)	Three modules: to-do list, individual health information, in-app chat service	Yes

^a^RCT: randomized controlled trial.

^b^BAS: before-after study.

^c^PHR: personal health record.

### Meta-Analysis

#### Pain Relief Rating

**Figure 3:**
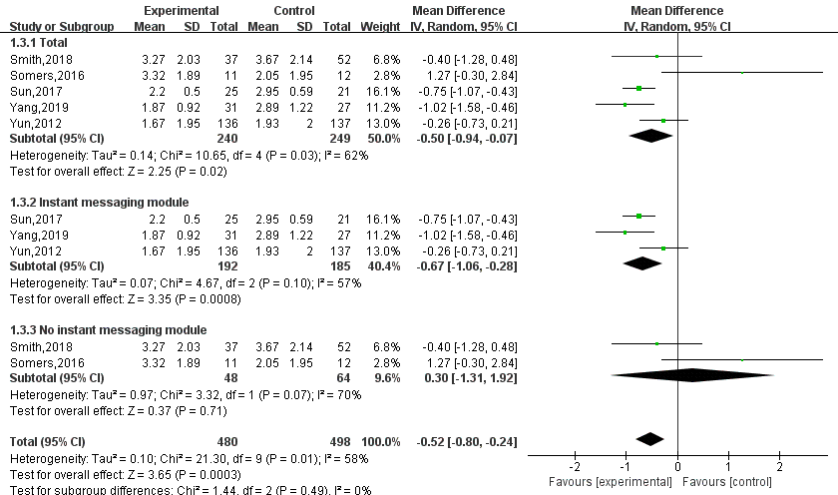
Subgroup analysis of the effects of apps with instant messaging modules on pain in patients with cancer.

A random-effects analysis model was used for the meta-analysis of the RCTs. The pain scores of the intervention group were lower than those of the control group (mean difference [MD]=–0.50, 95% CI –0.94 to –0.07, I^2^=62%, *P*=.02). Subgroup analysis was performed for apps with instant messaging modules. Instant messaging modules are a channel for real-time communication between patients and medical staff. Patients who used apps with instant messaging modules had lower pain scores (MD=–0.67, 95% CI –1.06 to –0.28, I^2^=57%, *P*<.01) than patients who used apps without an instant messaging module (MD=0.30, 95% CI –1.31 to 1.92, I^2^=70%, *P*=.71; Figure 3).

#### Sensitivity Analysis Results

The data were analyzed with the fixed- and random-effects model, and the consistency of these results reflects the reliability of the combined results to some extent. The two effect models were used to analyze the combined effect of each risk factor and calculate 95% CIs. The results were similar, indicating that the results of this study are stable.

### Impact of Apps on Other Outcomes

**Figure 4:**
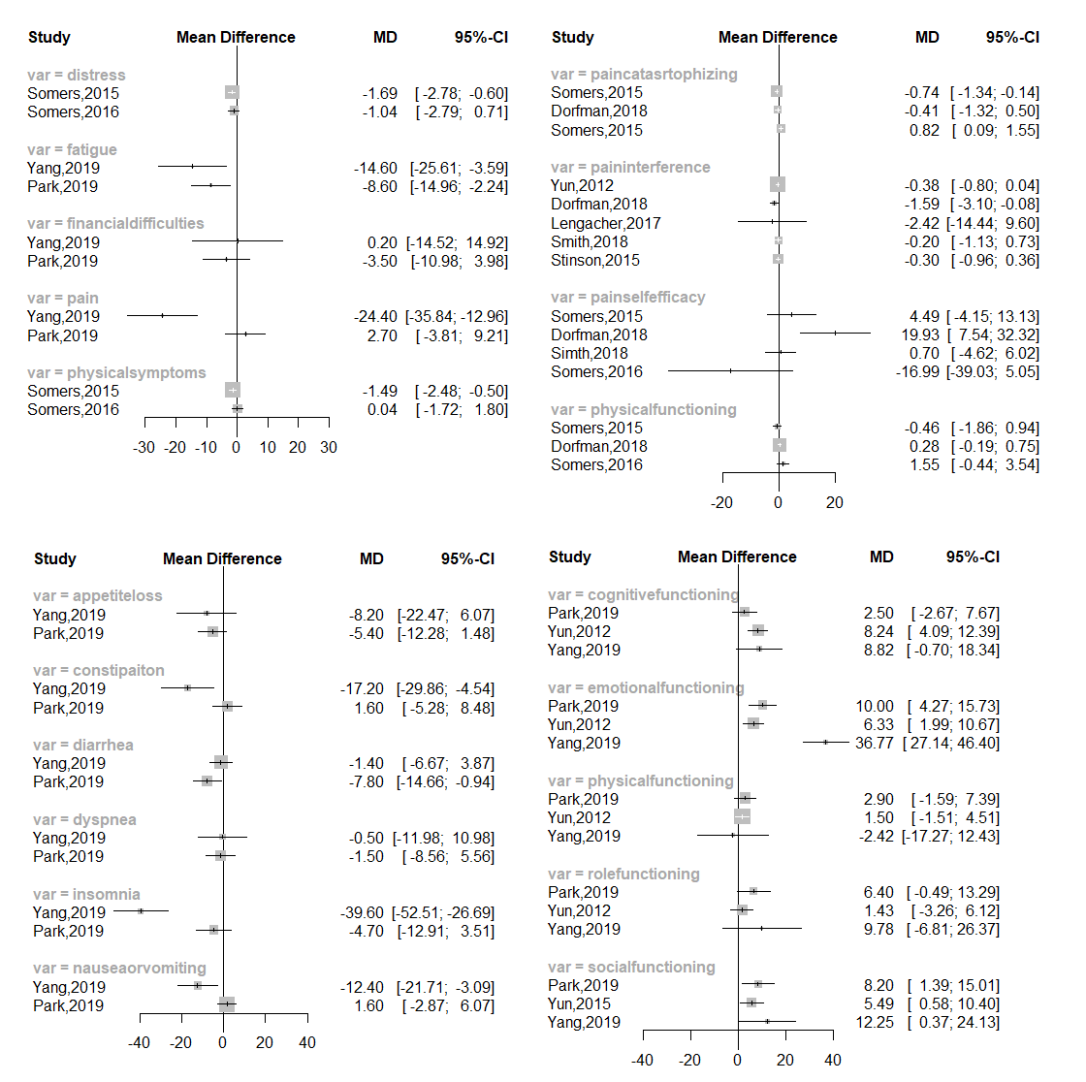
Forest plot of the effect of app use on other cancer-related outcomes.

Two studies reported that the number of side effects in patients using apps is statistically significantly lower than in the control group, whereas Bae et al reported no reduction in the number of adverse reactions [16,26]. All studies evaluating changes in quality of life and anxiety showed significant improvement in groups using apps [16,17,20,25,28]. In addition, in 3 studies, pain catastrophizing was lower, and pain self-efficacy was higher in the intervention group when compared to the control group [18,23]. However, Smith et al [28] reported no significant difference in pain catastrophizing or self-efficacy between the intervention and control groups, only a significant improvement in fatigue. Seven studies reported high levels of satisfaction with the ease of use of the app [16,18,20,21,25,28], and 5 studies showed that patients using apps had a higher intervention completion rate [18-20,25,28]. We created a forest plot to show the effects of apps on other outcomes (Figure 4). Most of the studies had no significant differences in results, apart from those for fatigue, emotional functioning, and social functioning.

## Discussion

### Principal Findings

The purpose of our study was to assess the effectiveness of apps for pain management in patients with cancer and to explore which modules influence pain relief ratings. A total of 13 studies were included in this study, and most of the studies reported that pain apps are effective. Patients who use the apps have less pain than patients who do not use the apps. Other results, such as quality of life, pain catastrophizing, and pain self-efficacy, significantly improved in patients using apps. Due to the lack of reported data and heterogeneity, a synthesis of the other results using statistical methods was not performed.

The apps used to manage cancer pain typically have multiple modules, including common records, training, and real-time feedback. These types of apps can be installed on a computer or mobile phone or accessed directly from a website. They can effectively teach patients about pain and provide the ability to record pain and provide feedback. To determine which modules have the greatest impact, we further conducted a subgroup analysis of the RCTs, which showed that, the use of an app that has instant messaging modules significantly improves pain relief ratings. Furthermore, the use of an app without instant messaging modules had no significant effect on the pain relief ratings of patients with cancer. Therefore, instant messaging modules in apps may be a key factor for pain relief. This may be because the patient can report their condition to the medical staff in a timely manner.

When using an app with an instant messaging module, patients with cancer can notify doctors or pharmacists of a pain outbreak or persistent pain, allowing pharmacists or doctors to intervene in real time. Additionally, a physician can give advice on the dosage of prescribed medication, manage the patient's pain and adverse drug reactions in real time, or recommend that the patient see a doctor immediately, thereby improving compliance with pain treatment and pain relief ratings [27,29]. A pharmacist can relieve stress by discussing how the patient is feeling or suggesting suitable alternative pain management methods. Studies have shown that yoga, proper exercise, or listening to soothing music can relieve pain [30,31]. These activities effectively allow patients to control their pain outside the hospital setting without panic or confusion [32]. Therefore, an instant messaging module could provide patients with both technical support and a sense of security when outside the hospital, which is an important part of pain management apps.

### Limitations

There are some limitations to our study. The included literature had relatively small sample sizes and varying follow-up times, some as short as 14 days. Despite these limitations, this study is the first to comprehensively analyze the effect of app modules on the ability of the app to effectively intervene. Therefore, larger samples and longer clinical RCTs are needed to further evaluate the impact of app intervention on pain relief ratings.

### Comparison With Prior Work

To the best of our knowledge, this is the first systematic review of the effectiveness of mobile apps for pain management in cancer patients. In published systematic reviews, the evaluation of the effects of apps focus on other chronic pain [6,13,33,34] or explore the impact of an app on quality of life and other symptoms for a single cancer type. They generally provide qualitative descriptions without an assessment of the overall effectiveness of the app as a tool for managing pain. These studies have also not identified which app modules are critical for the effectiveness of the app-based intervention [2,35].

The authors of 3 previous reviews reported that apps were not effective for the following reasons: The participation of health care workers was too low, the apps could not find resources in the online store, and the apps that could be downloaded did not pass scientific verification [14,36,37]. Rincon et al [2] pointed out that strict clinical trials are lacking for most apps. Three other reviews assessed multiple app types, which cannot quantitatively analyze the overall effectiveness of the apps on pain [35,38,39]. Martorella et al [38] and Fridriksdottir et al [39] studied the effects of multiple intervention types on pain, with a variety of intervention models, mainly based on the network that includes the app. Thurnheer et al [12] studied the benefits of an app for chronic pain. Of the 15 studies, 4 included in-hospital management, and 11 involved out-of-hospital management. Most preliminary reports suggest that an app is beneficial for relieving pain in patients, but all require additional scientific verification and analysis to determine which module is key for improving usability [12]. Silva et al [33] studied the effectiveness of self-management via an app for improving pain, psychological distress, fatigue, and sleep in cancer survivors. Only 6 studies were included, and the quality of the included literature was poor. Only 2 studies reported that the intervention group had a lower mean pain score at follow-up, but neither result reached statistical significance [33].

Other studies that did not meet our inclusion criteria (eg, studies that included patients with non-cancer pain and studies of different types) showed similar results to the studies included in our analysis. Schatz et al [40] used smartphone-based cognitive-behavioral therapy in children with sickle cell disease. The results suggested that smartphone-supported cognitive-behavioral therapy coping methods can reduce pain intensity in children with sickle cell disease. Sundberg et al [41] used an interactive smartphone app (Interaktor, Health Navigator, Sweden) to detect and treat symptoms during early prostate cancer radiotherapy, and the intervention group had significantly lower levels of fatigue and nausea at the end of radiotherapy. Therefore, it is an effective mobile health tool to promote supportive care during cancer treatment. Uhm et al [42] studied breast cancer patients using mobile health smartphone apps and pedometers, which resulted in greater improvements in quality of life than with traditional exercise manuals.

### Conclusions

This study found that patients with cancer might benefit from the use of apps for cancer pain management, especially those with instant messaging modules, which can reduce pain scores in cancer patients. Patient acceptance of these apps is high. At the same time, the app has a palliative effect on patients’ cancer pain and other cancer-related symptoms. Pain management apps with instant messaging modules provide the ability to connect patients and medical professionals with a convenient learning channel, especially in outpatient clinics and for patients living in remote areas with less access to doctors and medical support. Therefore, the findings of this study can guide the development of cancer pain apps, help optimize app modules, and narrow the gap between doctors and patients to achieve better control of cancer-related pain.

## Appendix

Multimedia Appendix 1 Search strategy to find literature on the use of apps for cancer pain management and interventions.

## Abbreviations

BAS: before-after study
MD: mean difference
PHR: personal health record
RCT: randomized controlled trial
