# Supplementary material for: Benefits of Mobile Apps for Cancer Pain Management: Systematic Review
Source: JMIR Mhealth Uhealth. 2020 Jan 23;8(1):e17055. doi: 10.2196/17055 (PMC7005688; doi:10.2196/17055)
Supplement: Multimedia Appendix 1 [file mhealth_v8i1e17055_app1.docx]

**Pubmed**

#1： (cancer pain[MeSH Terms]) OR (Pains, Neoplasm-Associated) OR (Oncological Pains) OR (Pain, Oncological) OR (Pains, Oncological) OR (Tumor-Related Pain Pain) OR (Tumor-Related) OR (Pains, Tumor-Related) OR (Tumor Related Pain) OR (Tumor-Related Pains) OR (Tumor-Associated Pain) OR (Pain, Tumor-Associated) OR (Pains, Tumor-Associated) OR (Tumor Associated Pain) OR (Tumor-Associated Pains) OR (Oncology Pain) OR (Oncology Pains) OR (Pain,Oncology) OR (Pains, Oncology) OR (Cancer-Related Pain) OR (Cancer Related Pain) OR (Cancer-Related Pains) OR (Pain, Cancer-Related) OR (Pains, Cancer-Related) OR (Neoplasm-Associated Pain) OR (Neoplasm Associated Pain) OR (Neoplasm-Associated Pains) OR (Pain, Neoplasm-Associated) OR (Oncological Pain) OR (Pain, Cancer) OR (Pains, Cance) OR (Cancer-Associated Pain) OR (Cancer Associated Pain) OR (Cancer-Associated Pains) OR (Pain, Cancer-Associated) OR (Pains, Cancer-Associated Neoplasm-Related Pain) OR (Neoplasm Related Pain) OR (Neoplasm-Related Pains) OR (Pain, Neoplasm-Related) OR (Pains, Neoplasm-Related) OR (pain[MeSH Terms]) OR (Injury) OR (Burning Pain) OR (Burning Pains) OR (Pains, Burning) OR (Suffering, Physical Radiating Pains) OR (Physical Suffering) OR (Physical Sufferings) OR (Sufferings, Physical) OR (Pain, Migratory) OR (Migratory Pain) OR (Migratory Pains) OR (Pains, Migratory) OR (Pain, Radiating) OR (Pains, Radiating) OR (Radiating Pain) OR (Pain, Splitting) OR (Pains, Splitting) OR (Splitting Pain) OR (Splitting Pains) OR (Ache) OR (Aches) OR (Pain, Crushing) OR (Crushing Pain) OR (Crushing Pains) OR (Pains, Crushing) OR (Pain, Burning) OR (Douleur) OR (Hurt) OR (Soreness) OR (Sore)

#2 ("Mobile Applications"[Mesh]) OR (Application, Mobile) OR (Applications, Mobile) OR (Mobile Application) OR (Mobile Apps App, Mobile) OR (Apps, Mobile) OR (Mobile App) OR (Portable Electronic Apps) OR (App, Portable Electronic) OR (Apps, Portable Electronic) OR (Electronic App, Portable) OR (Electronic Apps, Portable) OR (Portable Electronic App) OR (Portable Electronic Applications) OR (Application, Portable Electronic) OR (Applications, Portable Electronic) OR (Electronic Application, Portable) OR (Electronic Applications, Portable) OR (Portable Electronic Application) OR (Portable Software Apps) OR (App, Portable Software) OR (Apps, Portable Software) OR (Portable Software App) OR (Software App, Portable) OR (Software Apps, Portable) OR (Portable Software Applications) OR (Application, Portable Software) OR (Applications, Portable Software) OR (Portable Software Application) OR (Software Application, Portable) OR (Software Applications, Portable) OR (Mhealth[MeSH Terms]) OR (Mobile Health) OR (Health, Mobile) OR (mHealth) OR (Telehealth) OR (eHealth)

#3：#1 or #2

**Embase**

**#1：**'pain'/exp OR 'injury' OR 'burning pain' OR 'burning pains' OR 'pains, burning' OR 'suffering, physical' OR 'physical suffering' OR 'physical sufferings' OR 'sufferings, physical' OR 'pain, migratory' OR 'migratory pain' OR 'migratory pains' OR 'pains, migratory' OR 'pain, radiating' OR 'pains, radiating' OR 'radiating pain' OR 'radiating pains' OR 'pain, splitting' OR 'pains, splitting' OR 'splitting pain' OR 'splitting pains' OR 'ache' OR 'aches' OR 'pain, crushing' OR 'crushing pain' OR 'crushing pains' OR 'pains, crushing' OR 'pain, burning' OR 'douleur' OR 'hurt' OR 'soreness' OR 'sore' OR 'cancer pain'/exp OR 'primary cancer pain' OR 'cancer pains' OR 'pain, cancer' OR 'pains, cancer' OR 'cancer-associated pain' OR 'cancer associated' OR 'pain cancer-associated pains' OR 'pain, cancer-associated' OR 'pains, cancer-associated' OR 'neoplasm-related pain' OR 'neoplasm related pain' OR 'neoplasm-related pains' OR 'pain, neoplasm-related' OR 'pains, neoplasm-related' OR 'oncological pain' OR 'oncological pains' OR 'pain, oncological' OR 'pains, oncological' OR 'tumor-related pain' OR 'pain, tumor-related' OR 'pains, tumor-related' OR 'tumor related pain' OR 'tumor-related pains' OR 'tumor-associated pain' OR 'pain, tumor-associated' OR 'pains, tumor-associated' OR 'tumor associated pain' OR 'tumor-associated pains' OR 'oncology pain' OR 'oncology pains' OR 'pain, oncology' OR 'pains, oncology' OR 'cancer-related pain' OR 'cancer related pain' OR 'cancer-related pains' OR 'pain, cancer-related' OR 'pains, cancer-related' OR 'neoplasm-associated pain' OR 'neoplasm associated pain' OR 'neoplasm-associated pains' OR 'pain, neoplasm-associated' OR 'pains, neoplasm-associated' OR 'cancerous pain'

#2：'application, mobile' OR (application, AND mobile) OR 'applications, mobile' OR (applications, AND mobile) OR 'mobile application'/exp OR 'mobile application' OR (mobile AND application) OR 'mobile apps app, mobile' OR (apps AND app, AND mobile) OR 'apps, mobile' OR (apps, AND mobile) OR 'mobile app'/exp OR 'mobile app' OR (mobile AND app) OR 'portable electronic apps' OR (portable AND electronic AND apps) OR 'app, portable electronic' OR (app, AND portable AND electronic) OR 'apps, portable electronic' OR (apps, AND portable AND electronic) OR 'electronic app, portable' OR (electronic AND app, AND portable) OR 'electronic apps, portable' OR (electronic AND apps, AND portable) OR 'portable electronic app' OR (portable AND electronic AND app) OR 'portable electronic applications' OR (portable AND electronic AND applications) OR 'application, portable electronic' OR (application, AND portable AND electronic) OR 'applications, portable electronic' OR (applications, AND portable AND electronic) OR 'electronic application, portable' OR (electronic AND application, AND portable) OR 'electronic applications, portable' OR (electronic AND applications, AND portable) OR 'portable electronic application' OR (portable AND electronic AND application) OR 'portable software apps'/exp OR 'portable software apps' OR (portable AND ('software'/exp OR software) AND apps) OR 'app, portable software' OR (app, AND portable AND ('software'/exp OR software)) OR 'apps, portable software' OR (apps, AND portable AND ('software'/exp OR software)) OR 'portable software app'/exp OR 'portable software app' OR (portable AND ('software'/exp OR software) AND app) OR 'software app, portable' OR (('software'/exp OR software) AND app, AND portable) OR 'software apps, portable' OR (('software'/exp OR software) AND apps, AND portable) OR 'portable software applications'/exp OR 'portable software applications' OR (portable AND ('software'/exp OR software) AND applications) OR 'application, portable software' OR (application, AND portable AND ('software'/exp OR software)) OR 'applications, portable software' OR (applications, AND portable AND ('software'/exp OR software)) OR 'portable software application'/exp OR 'portable software application' OR (portable AND ('software'/exp OR software) AND application) OR 'software application, portable' OR (('software'/exp OR software) AND application, AND portable) OR 'software applications, portable' OR (('software'/exp OR software) AND applications, AND portable) OR 'Mhealth'/exp OR 'Mobile Health' OR 'Health, Mobile' OR 'mHealth' OR 'Telehealth' OR 'eHealth'

#3: #1 and #2

[**Cochrane Library**](http://www.baidu.com/link?url=ajG4RuqShux6nlg5oTRROhYeepvQXFzvRL5tshPuX6zmvs4bhDWv9Hs5W1lTPi3B8inUJlqu2pk7fz2CClGFRESr255lRCshyLvCoiYaF2S)

#1 Pain[mh] or (Burning Pain) or (Burning Pains) or (Pains, Burning) or (Suffering, Physical) or (Physical Suffering) or (Physical Sufferings) or (Sufferings, Physical) or (Pain, Migratory) or (Migratory Pain) or (Migratory Pains) or (Pains, Migratory) or (Pain, Radiating) or (Pains, Radiating) or (Radiating Pain) or (Radiating Pains) or (Pain, Splitting) or (Pains, Splitting) or (Splitting Pain) or (Splitting Pains) or (Ache) or (Aches) or (Pain, Crushing) or (Crushing Pain) or (Crushing Pains) or (Pains, Crushing) or (Pain, Burning) or (Douleur) or (Hurt) or (Soreness) or (Sore) or (Injury) or Cancer Pain[mh] or (Cancer Pains) or (Pain, Cancer) or (Pains, Cancer) or (Cancer-Associated Pain) or (Cancer Associated Pain) or (Cancer-Associated Pains) or (Pain, Cancer-Associated) or (Pains, Cancer-Associated) or (Neoplasm-Related Pain) or (Neoplasm Related Pain) or (Neoplasm-Related Pains) or (Pain, Neoplasm-Related) or (Pains, Neoplasm-Related) or (Oncological Pain) or (Oncological Pains) or (Pain, Oncological) or (Pains, Oncological) or (Tumor-Related Pain) or (Pain, Tumor-Related) or (Pains, Tumor-Related) or (Tumor Related Pain) or (Tumor-Related Pains) or (Tumor-Associated Pain) or (Pain, Tumor-Associated) or (Pains, Tumor-Associated) or (Tumor Associated Pain) or (Tumor-Associated Pains) or (Oncology Pain) or (Oncology Pains) or (Pain, Oncology) or (Pains, Oncology) or (Cancer-Related Pain) or (Cancer Related Pain) or (Cancer-Related Pains) or (Pain, Cancer-Related) or (Pains, Cancer-Related) or (Neoplasm-Associated Pain) or (Neoplasm Associated Pain) or (Neoplasm-Associated Pains) or (Pain, Neoplasm-Associated) or (Pains, Neoplasm-Associated) or (cancerous pain) or (primary cancer pain)

#2 Mobile Applications[mh] or (Application, Mobile) or (Applications, Mobile) or (Mobile Application) or (Mobile Apps) or (App, Mobile) or (Apps, Mobile) or (Mobile App) or (Portable Electronic Apps) or (App, Portable Electronic) or (Apps, Portable Electronic) or (Electronic App, Portable) or (Electronic Apps, Portable) or (Portable Electronic App) or (Portable Electronic Applications) or (Application, Portable Electronic) or (Applications, Portable Electronic) or (Electronic Application, Portable) or (Electronic Applications, Portable) or (Portable Electronic Application) or (Portable Software Apps) or (App, Portable Software) or (Apps, Portable Software) or (Portable Software App) or (Software App, Portable) or (Software Apps, Portable) or (Portable Software Applications) or (Application, Portable Software) or (Applications, Portable Software) or (Portable Software Application) or (Software Application, Portable) or (Software Applications, Portable) or [Telemedicine](https://www-cochranelibrary-com.ezproxymcp.flo.org/advanced-search/mesh#0)[mh] or (Mhealth) or (Mobile Health) or (Health, Mobile) or (mHealth) or (Telehealth) or (eHealth)

#3 #1 and #2

**CINAHL**

#1: (MM " Cancer Pain ") OR(Pains, Neoplasm-Associated) OR (Oncological Pains) OR (Pain, Oncological) OR (Pains, Oncological) OR (Tumor-Related Pain Pain) OR (Tumor-Related) OR (Pains, Tumor-Related) OR (Tumor Related Pain) OR (Tumor-Related Pains) OR (Tumor-Associated Pain) OR (Pain, Tumor-Associated) OR (Pains, Tumor-Associated) OR (Tumor Associated Pain) OR (Tumor-Associated Pains) OR (Oncology Pain) OR (Oncology Pains) OR (Pain,Oncology) OR (Pains, Oncology) OR (Cancer-Related Pain) OR (Cancer Related Pain) OR (Cancer-Related Pains) OR (Pain, Cancer-Related) OR (Pains, Cancer-Related) OR (Neoplasm-Associated Pain) OR (Neoplasm Associated Pain) OR (Neoplasm-Associated Pains) OR (Pain, Neoplasm-Associated) OR (Oncological Pain) OR (Pain, Cancer) OR (Pains, Cance) OR (Cancer-Associated Pain) OR (Cancer Associated Pain) OR (Cancer-Associated Pains) OR (Pain, Cancer-Associated) OR (Pains, Cancer-Associated Neoplasm-Related Pain) OR (Neoplasm Related Pain) OR (Neoplasm-Related Pains) OR (Pain, Neoplasm-Related) OR (Pains, Neoplasm-Related) OR (MM " Pain+") OR(Injury) OR (Burning Pain) OR (Burning Pains) OR (Pains, Burning) OR (Suffering, Physical Radiating Pains) OR (Physical Suffering) OR (Physical Sufferings) OR (Sufferings, Physical) OR (Pain, Migratory) OR (Migratory Pain) OR (Migratory Pains) OR (Pains, Migratory) OR (Pain, Radiating) OR (Pains, Radiating) OR (Radiating Pain) OR (Pain, Splitting) OR (Pains, Splitting) OR (Splitting Pain) OR (Splitting Pains) OR (Ache) OR (Aches) OR (Pain, Crushing) OR (Crushing Pain) OR (Crushing Pains) OR (Pains, Crushing) OR (Pain, Burning) OR (Douleur) OR (Hurt) OR (Soreness) OR (Sore)

#2:(MM "Mobile Applications") OR(Application, Mobile) OR (Applications, Mobile) OR (Mobile Application) OR (Mobile Apps App, Mobile) OR (Apps, Mobile) OR (Mobile App) OR (Portable Electronic Apps) OR (App, Portable Electronic) OR (Apps, Portable Electronic) OR (Electronic App, Portable) OR (Electronic Apps, Portable) OR (Portable Electronic App) OR (Portable Electronic Applications) OR (Application, Portable Electronic) OR (Applications, Portable Electronic) OR (Electronic Application, Portable) OR (Electronic Applications, Portable) OR (Portable Electronic Application) OR (Portable Software Apps) OR (App, Portable Software) OR (Apps, Portable Software) OR (Portable Software App) OR (Software App, Portable) OR (Software Apps, Portable) OR (Portable Software Applications) OR (Application, Portable Software) OR (Applications, Portable Software) OR (Portable Software Application) OR (Software Application, Portable) OR (Software Applications, Portable) OR (MM "Telehealth+") OR (Mobile Health) OR (Health, Mobile) OR (mHealth) OR (Telehealth) OR (eHealth)

#3:#1 and #2

**PsycINFO**

#1:(MM "Mobile Applications") OR(Application, Mobile) OR (Applications, Mobile) OR (Mobile Application) OR (Mobile Apps App, Mobile) OR (Apps, Mobile) OR (Mobile App) OR (Portable Electronic Apps) OR (App, Portable Electronic) OR (Apps, Portable Electronic) OR (Electronic App, Portable) OR (Electronic Apps, Portable) OR (Portable Electronic App) OR (Portable Electronic Applications) OR (Application, Portable Electronic) OR (Applications, Portable Electronic) OR (Electronic Application, Portable) OR (Electronic Applications, Portable) OR (Portable Electronic Application) OR (Portable Software Apps) OR (App, Portable Software) OR (Apps, Portable Software) OR (Portable Software App) OR (Software App, Portable) OR (Software Apps, Portable) OR (Portable Software Applications) OR (Application, Portable Software) OR (Applications, Portable Software) OR (Portable Software Application) OR (Software Application, Portable) OR (Software Applications, Portable) OR (MM "mobile health") OR (Mobile Health) OR (Health, Mobile) OR (mHealth) OR (Telehealth) OR (eHealth)

2#:"( MM "Pain" OR MM "Aphagia" OR MM "Back Pain" OR MM "Chronic Pain" OR MM "Headache" OR MM "Myofascial Pain" OR MM "Neuralgia" OR MM "Neuropathic Pain" OR MM "Somatoform Pain Disorder" ) OR ( (Injury) OR (Burning Pain) OR (Burning Pains) OR (Pains, Burning) OR (Suffering, Physical Radiating Pains) OR (Physical Suffering) OR (Physical Sufferings) OR (Sufferings, Physical) OR (Pain, Migratory) OR (Migratory Pain) OR (Migratory Pains) OR (Pains, Migratory) OR (Pain, Radiating) OR (Pains, Radiating) OR (Radiating Pain) OR (Pain, Splitting) OR (Pains, Splitting) OR (Splitting Pain) OR (Splitting Pains) OR (Ache) OR (Aches) OR (Pain, Crushing) OR (Crushing Pain) OR (Crushing Pains) OR (Pains, Crushing) OR (Pain, Burning) OR (Douleur) OR (Hurt) OR (Soreness) OR (Sore) )

**Scopus**

( ( cancer pain[mesh terms] ) OR (pains, AND neoplasm-associated) OR (oncological AND pains) OR (pain, AND oncological ) OR ( pains, AND oncological ) OR ( tumor-related AND pain AND pain ) OR ( tumor-related ) OR ( pains, AND tumor-related ) OR ( tumor AND related AND pain ) OR ( tumor-related AND pains ) OR ( tumor-associated AND pain ) OR ( pain, AND tumor-associated ) OR ( pains, AND tumor-associated ) OR ( tumor AND associated AND pain ) OR ( tumor-associated AND pains ) OR ( oncology AND pain ) OR ( oncology AND pains ) OR ( pain,oncology ) OR ( pains, AND oncology ) OR ( cancer-related AND pain ) OR ( cancer AND related AND pain ) OR ( cancer-related AND pains ) OR ( pain, AND cancer-related ) OR ( pains, AND cancer-related ) OR ( neoplasm-associated AND pain ) OR ( neoplasm AND associated AND pain ) OR ( neoplasm-associated AND pains ) OR ( pain, AND neoplasm-associated ) OR ( oncological AND pain ) OR ( pain, AND cancer ) OR ( pains, AND cance ) OR ( cancer-associated AND pain ) OR ( cancer AND associated AND pain ) OR ( cancer-associated AND pains ) OR ( pain, AND cancer-associated ) OR ( pains, AND cancer-associated AND neoplasm-related AND pain ) OR ( neoplasm AND related AND pain ) OR ( neoplasm-related AND pains ) OR ( pain, AND neoplasm-related ) OR ( pains, AND neoplasm-related ) ) AND ( ( "Mobile Applications" [mesh] ) OR ( application, AND mobile ) OR ( applications, AND mobile ) OR ( mobile AND application ) OR ( mobile AND apps AND app, AND mobile ) OR ( apps, AND mobile ) OR ( mobile AND app ) OR ( portable AND electronic AND apps ) OR ( app, AND portable AND electronic ) OR ( apps, AND portable AND electronic ) OR ( electronic AND app, AND portable ) OR ( electronic AND apps, AND portable ) OR ( portable AND electronic AND app ) OR ( portable AND electronic AND applications ) OR ( application, AND portable AND electronic ) OR ( applications, AND portable AND electronic ) OR ( electronic AND application, AND portable ) OR ( electronic AND applications, AND portable ) OR ( portable AND electronic AND application ) OR ( portable AND software AND apps ) OR ( app, AND portable AND software ) OR ( apps, AND portable AND software ) OR ( portable AND software AND app ) OR ( software AND app, AND portable ) OR ( software AND apps, AND portable ) OR ( portable AND software AND applications ) OR ( application, AND portable AND software ) OR ( applications, AND portable AND software ) OR ( portable AND software AND application ) OR ( software AND application, AND portable ) OR ( software AND applications, AND portable ) OR ( mhealth[mesh AND terms] ) OR ( mobile AND health ) OR ( health, AND mobile ) OR ( mhealth ) OR ( telehealth ) OR ( ehealth ) )
